# Adjuvant Treatment for Protocadherin 19 (PCDH19) Syndrome

**DOI:** 10.7759/cureus.27154

**Published:** 2022-07-22

**Authors:** Juan A Moncayo, Maite N Vargas, Isabel Castillo, Pablo V Granda, Andrea M Duque, Jennifer M Argudo, Sakina Matcheswalla, Guillermo E Lopez Dominguez, Gustavo Monteros, Andres F Andrade, Diego Ojeda, Mario Yepez

**Affiliations:** 1 Neurology, Pontificia Universidad Católica del Ecuador, Quito, ECU; 2 Neurology, Universidad San Francisco de Quito, Quito, ECU; 3 Medicine, Universidad San Francisco de Quito, Quito, ECU; 4 Medicine, Pontificia Universidad Católica del Ecuador, Quito, ECU; 5 General Medicine, Pontificia Universidad Católica del Ecuador, Quito, ECU; 6 Child Neurology, Universidad de Cuenca, Cuenca, ECU; 7 Neurology, Krishna Institute of Medical Science, Mumbai, IND; 8 Medicine, Universidad de Cuenca, Cuenca, ECU; 9 Neurology, Universidad San Fracisco de Quito, Quito, ECU; 10 Neurology, Universidad Católica Santiago de Guayaquil, Guayaquil, ECU

**Keywords:** ganaxolone, clobazam, stiripentol, refractory epileps, epilepsy

## Abstract

Protocadherin 19 (PCDH19) syndrome is inherited as an X-linked pattern and affects mainly females. This syndrome is caused by a mutation in the PCDH19 gene encoding for the protocadherin protein. It is characterized by refractory seizures during febrile episodes with neuropsychiatric manifestations. There is no consensus on the treatment of PCDH19. We conducted a literature review to investigate the main drugs used for this syndrome, and to evaluate the best possible course of adjuvant treatment for these patients. We used an advanced PubMed search strategy with the following inclusion criteria: a) full-text papers, b) English Language, and c) studies conducted in humans. Exclusion criteria: a) literature reviews, b) systematic reviews, and c) metanalysis. We gathered 26 observational papers to conduct this literature review on clobazam and bromide which have been shown to reduce seizures by 50%. Corticosteroids improved neurological symptoms during the episodes in a few patients. Nevertheless, they recurred after a few months. Preliminary results of ganaxolone, which is still under study, demonstrated a reduction of 60% in seizure episodes. A ketogenic diet has been studied to treat several refractory epilepsies, including PCDH19; it has promising results as effective adjuvant therapy in the resolution of status epilepticus, suggesting it could be used as part of the treatment in early childhood. Stiripentol was given as adjuvant therapy in a patient with PCDH19 epilepsy resulting in the most extended period of seizure-free episodes, but more studies must be performed to assess its efficacy.

## Introduction and background

Protocadherin 19 (PCDH19) syndrome is a type of epilepsy related to the PCDH19 gene (X-linked), which encodes for a protein that seems to regulate gamma-aminobutyric acid type A receptors (GABAAR). This adhesion molecule, called protocadherin-19, induces GABAergic signaling to promote the differentiation of neuronal progenitors, neuronal migration, and maturation [1].

The seizures can be tonic-clonic, atonic, myoclonic, or absent. Typically, seizures in PCDH19 present in clusters, which is defined as having two or three seizures within 24 hours with recovery between seizures [2]. The onset of seizures occurs between 10 and 14 months of age and may decrease or go into complete remission in adolescence. A high proportion of patients with this affliction also present with neuropsychiatric manifestations, the most common being developmental delays, autistic traits, and behavioral abnormalities. The syndrome affects mostly females, despite being X-linked due to silencing an X chromosome in heterozygote females [3].

Anti-epileptic drugs (AEDs) are the first line of treatment used to prevent seizures and stop seizure clusters, even though there are limited reports of medications for PCDH19 epilepsy, and it is difficult to assess the effectiveness of antiepileptic drugs in these patients [4]. The treatment of PCDH19-epilepsy syndrome is difficult as seizures are highly resistant to anti-epileptic drugs in the early years of life, although this pattern inverts as the patient grows older [4-6]. Early initiation of AEDs decreases the overall seizure load, which is important because seizure load is indirectly linked to poor cognitive outcomes [5]. The efficacy of some AEDs is still being researched. Phenytoin, potassium bromide, and clobazam are effective in decreasing seizure events, but the duration of their effects is transient [5,7]. This literature review aims to assess adjuvant treatments other than the classic AEDs commonly used in clinical practice as supplemental aids to clinical practice.

## Review

Materials and methods

We used a PubMed search strategy with the following inclusion criteria: a) full-text papers, b) English language, and c) studies conducted in humans. Exclusion criteria were: a) literature reviews, b) systematic reviews, and c) metanalysis. We use the following advanced search terms: [.1] (PCDH19 [Title/Abstract]) AND (ganaxolone [Title/Abstract]); (“PCDH19” [Title/Abstract] AND “steroids” [Title/Abstract]) OR (“PCDH19” [Title/Abstract] AND “corticosteroids” [Title/Abstract]) ; (“PCDH19” [Title/Abstract] AND “stiripentol” [Title/Abstract]); (“PCDH19” [Title/Abstract] AND “clobazam and potassium bromide” [Title/Abstract]); (“PCDH19” [Title/Abstract] AND “ketogenic diet” [Title/Abstract]).

Results

Table 1 below shows the results gathered from the advanced PubMed Search.

**Table 1:** Advanced PubMed Search PCDH19: Protocadherin 19

Search Terms	Results
(PCDH19[Title/Abstract]) AND (Ganaxolone [Title/Abstract])	4
("PCDH19"[Title/Abstract] AND "Steroids"[Title/Abstract]) OR ("PCDH19"[Title/Abstract] AND "Corticosteroids"[Title/Abstract])	8
(PCDH19[Title/Abstract]) AND (Stiripentol [Title/Abstract])	3
(PCDH19[Title/Abstract]) AND (Ketogenic Diet [Title/Abstract])	4
((Clobazam [Title/Abstract]) OR (Bromide[Title/Abstract])) AND (PCDH19[Title/Abstract])	6

Discussion

Unfortunately, PCDH19 is usually refractory to antiepileptic treatment. It is difficult to judge the effectiveness of any antiepileptic drug since the syndrome is variable, and seizures can occur with fever. As opposed to Dravet syndrome, PCDH19 seizures are not triggered by the use of sodium channel blockers [8].

The seizures associated with PCDH19 are more resistant during early childhood, however, the frequency and intractability of seizures tend to decrease over time [9]. A certain percentage of patients are seizure free during adolescence or adult life [9]. Status epilepticus is a common occurrence in these patients, and benzodiazepines are commonly used during these episodes [10].

In a study by Lotte et al., after receiving more than three medications, 78% of patients became seizure-free for at least three months, and 45% achieved long-term, seizure-free remission for more than 24 months [4]. Only 3% of patients in the same study achieved seizure-free status for more than 10 years. Assessing the effectiveness of these drugs is challenging because these specific seizures are age-dependent. Nevertheless, 46% of these subjects were younger than five years old [4].

We assessed adjuvant treatments used in observational studies for PCDH19 refractory epilepsy and reviewed its mechanisms of action and effectiveness.

Clobazam & Potassium Bromide

Clobazam is a benzodiazepine with a unique structure in that it has different nitrogen radicals from other benzodiazepines, allowing it to be an effective anticonvulsant [9]. Currently, it is used mainly to treat epileptic seizures [10].

Potassium bromide, which activates GABAR, was the first antiepileptic drug to treat epilepsy in the 19th century. Its use was discontinued after the discovery of phenobarbital in the 1910s. Currently, with reports of its efficacy in refractory epilepsy in the 1990s, potassium bromide is attracting attention [11-12]. In a case study by Higurashi et al., Asian patients with deletion of the PCDH19 gene showed a satisfactory response to the prophylactic use of potassium bromide, phenytoin, and clobazam to reduce seizure frequency [7].

In a multicenter study by Lotte et al., which was conducted in 25 hospitals in 12 different countries with a population of 58 patients, clobazam and bromide were the most effective treatments, with the latter being considered the most efficient during the 12 months of the study. The response rate to clobazam and bromide at three months was 68% and 67%, respectively; the response was defined as a seizure reduction of at least 50%. Contrary to what has been seen in the treatment of other types of seizures, the effect of clobazam in these patients was not lost over time [4].

It is difficult to quantify the response to treatment in patients with PCDH19 syndrome since, in addition to its low incidence and prevalence, it tends to present as a spectrum of symptoms and signs. This adds to the natural history of this disease, which has been shown to decrease seizure frequency and intensity as the patient ages [4]. Still, due to the similarity of the pathophysiology of this disease with Dravet syndrome, drugs with a GABA receptor agonist mechanism are helpful. The use of clobazam together with valproate and stiripentol, a pharmacological combination used for the treatment of Dravet syndrome, has shown favorable results in a case report of PCDH19 by Trivisano et al. [13]. 

Corticosteroids

Corticosteroids are believed to function as a treatment for this disease since one of the pathogenic mechanisms of the disease is an autoimmune inflammation of the blood-brain barrier (BBB). The BBB is believed to be involved in the generation of seizures based on the fact that anti-NMDA receptor antibodies (abs-NR) and anti-neuronal antibodies have been found in the cerebrospinal fluid (CSF) of subjects [14]. Likewise, PHCD19, which is highly expressed in the endothelial cells of the BBB, is probably modified during inflammation of the BBB by abs-NR, therefore predisposing patients to seizure episodes [15].

Daneman et al. purified endothelial cells from the BBB of transgenic mice using fluorescence-activated cell sorting to determine the development of the BBB and the function of its endothelial cells. Most of them expressed green fluorescent protein, Platelet endothelial cell adhesion molecule-1 (PECAM1), claudin5, and occludins. In addition, signaling pathways in the BBB were identified, including the Wnt/beta-catenin signaling pathway, which has been demonstrated to be highly activated in the central nervous system (CNS) of the endothelial barrier, functioning to regulate angiogenesis and the expression of tight junctions. Another recognized pathway in the CNS endothelial cells is the lipopolysaccharide (LPS)/interleukin-1 (IL-1)-mediated inhibition of the retinoic-X receptor, which can also be inhibited by IL-1, LPS, or tumor necrosis factor-alpha (TNF-α) [15], which are all recognized pro-inflammatory cytokines. These findings could support the use of corticosteroids in the management of PCDH19 syndrome.

Salvador et al. proposed another mechanism for the reduction of edema through the restoration of tight junctions in BBB endothelial cells, where Higurashi et al. found an increased expression of PCDH19 [14,16]. The subjects in Salvador et al.’s study showed great improvement in their neurological symptoms after the first dose of glucocorticoids. Moreover, some patients were treated with prophylactic doses for three days with 0.01mg/kg of oral betamethasone or 1 to 1.5 mg/kg of oral prednisolone after the age of three every time they experienced an episode of fever and showed a decrease in the recurrence of seizures and neurological manifestations [14]. In the case study by Higurashi et al., the early administration of 10 to 30 mg/kg of intravenous methylprednisolone in one patient while the other one received an intravenous infusion of 0.35mg/kg of prednisolone followed by a dose of 1mg/kg/day. Both patients with risk factors resulted in a transient resolution of seizures occurring at that moment [7]. 

Ganaxolone

A synthetic analog of endogenous allopregnanolone, ganaxolone modulates GABAR as a positive allosteric modulator in the CNS. It has been studied for the management of some refractory epilepsies, including PCDH19-related epilepsy. Its use was approved by the Food and Drug Administration (FDA) for seizure disorders with cyclin-dependent kinase 5 (CDKK5) enzyme deficiency in patients older than two years.

Currently, an ongoing double-blind, randomized, placebo-controlled clinical trial called the Violet study (phase 2) is being conducted with 25 female patients with a confirmed PCDH19 mutation. The subjects received either ganaxolone or a placebo for 17 weeks. The primary outcome of the study was to establish the percentage of reduction in seizure frequency in 28 days. The study will be completed in mid-2022. So far, data obtained after treatment lasting 17 weeks have shown a reduction in seizures of more than 60% in the experimental group compared to 24% in the placebo group. Regarding the safety of this medication, more than two-thirds of the patients had associated adverse effects that would have been reported. Despite this encouraging result, more clinical trials are necessary before considering this drug as a safe alternative treatment for PCDH19 syndrome [17].

Ketogenic Diet

The ketogenic diet involves reducing the intake of carbohydrates to a minimum and increasing protein and fat intake, which shifts the body into a metabolic state called ketosis, in which the body uses fats as a primary fuel source [18].

The ketogenic diet was first used in 1920 to treat resistant epilepsy [19]. Sampaio et al. proposed that the mechanism lies in increasing GABA synthesis and decreasing the release of glutamate and norepinephrine [18]. Wang et al. described an increased amount of GABA in the CSF of patients on a ketogenic diet. This diet has been used in pediatric patients with super-refractory seizure disorders, showing partial or total reduction of seizures within one to 19 days after patients started the diet [20]. This diet stimulates the opening of adenosine triphosphate-sensitive potassium (K-ATP) channels [21] and promotes the presynaptic accumulation of GABA. Musa et al. claim that diet alone cannot reach therapeutic levels and suggest that a ketogenic diet must be used in combination with AEDs [22].

A retrospective, multicenter study conducted by Caraballo et al. in Argentina described the efficacy and tolerability of a ketogenic diet in patients with different types of epilepsy, including PCDH19 syndrome, aged one to 18 years. The study found that 31 of the 140 patients who remained on a ketogenic diet became seizure-free, and 48 had a decrease in the number of seizures. Among the five patients diagnosed with febrile infection-related epilepsy, only one became seizure-free, and 10 out of the 31 patients who became seizure-free were treated in combination with other AEDs. The most common adverse effects were all treatable, the most common being vomiting and diarrhea [22].

Appavu et al. treated 10 patients diagnosed with super-refractory status epilepticus. The PCDH19 mutation was detected in only one of these patients. Treatment with levetiracetam, clobazam, and carbamazepine and, as adjuvant therapy, a ketogenic diet resulted in a decrease in the frequency of seizures in one patient with PCDH19 and eight other patients with other types of epilepsy [23].

Stiripentol

Although stiripentol’s mechanism of action is not completely understood, it is believed that it enhances the inhibitory effect of the GABA neurotransmitter by behaving as a partial agonist or as an allosteric modulator of its receptor in the postsynaptic membrane [24-26]. In addition, it affects the processes mediated by cytochrome P450, which include the metabolism of AEDs, triggering an increase in their bioavailability and a delay in their excretion from the body [27].

Currently, there is no pharmacological treatment for PCDH19. However, Dravet syndrome, childhood epilepsy characterized by drug-resistant seizures and episodes induced by fever such as PCDH19, was approved in 2018 as part of the second-line drug regimen. Among its effects, encouraging findings stand out, such as a reduction in the duration of seizures and decreased frequency of hospitalizations. Observational studies have found that these effects persist even after patients reach adulthood [28]. It is recommended that stiripentol be administered with other AEDs (such as valproate); there is no evidence of satisfactory results if used as a monotherapy [13].

Over time, attempts have been made to use this drug to treat other types of seizure disorders, with both encouraging and inconclusive results. In 2014, it was added to the medication regimen administered to a 12-year-old girl diagnosed with PCDH-19-related epilepsy who was already being treated with valproate and clobazam [13]. The most striking effect of this case report is that, following the addition of stiripentol, the patient experienced her longest-ever seizure-free period. It is unknown whether this was due to the partial agonism of this medication toward inhibitory neurotransmitters or due to an increase in the concentration of the other drugs caused by stiripentol itself [13].

Behavioral and Psychiatric Role

Many children diagnosed with epilepsy suffer from a broad spectrum of neuropsychiatric comorbidities, the most common of which are attention deficits, mental slowness, and memory impairments [29]. Several factors intercorrelate and contribute to cognitive impairment, but only three have been confirmed: electroclinical seizures, the etiology of epilepsy, and the side effects of AEDs in the central nervous system [30].

As determined by Kim et al., there is a clear relationship between epilepsy onset, severity, and cognitive decline. In PCDH19 and Dravet syndrome, it has been well demonstrated that the duration and frequency of seizures directly impact brain development, demonstrating the importance of early and effective treatment, which can have a significant impact on prognosis [31]. However, these kinds of refractory epilepsies are often not well controlled, so early identification provides a window to introduce developmental interventions, vocational counseling, appropriate school programing, supportive work settings, and promotion of independence [30].

Vlaskamp et al. studied the incidence of psychiatric disorders as a late manifestation in female patients with PCDH19 syndrome and found that more than 10% of a group of patients had behavioral problems and psychosis. This condition presented as a spectrum of psychiatric illnesses that included schizophrenia, schizoaffective disorder, and nonspecific psychotic disorder. A higher incidence was reported in patients with more intellectual disabilities. It is believed that for this reason, patients diagnosed with PCDH19 should be monitored closely and treated promptly to improve their quality of life [31].

The behavioral and psychiatric features associated with PCDH19 epilepsy must be managed by behavioral health professionals. Similarly, cognitive and behavioral delays should be managed by speech, occupational, and physical therapists. Moreover, family support and training are crucial parts of epilepsy management. 

## Conclusions

Our objective was to review adjuvant treatments other than the most common AEDs used for PCDH19 syndrome. Although these treatments have resulted in a reduction in seizures, most of the patients in the studies highlighted were already on a multiple-drug regimen. Of the interventions studied, the most effective were clobazam and bromide, which reduced the rate of seizures by more than half. A ketogenic diet, which decreases neuronal hyperexcitability by increasing membrane potential hyperpolarization, decreased the number of seizures reported. Ganaxolone is a new drug that resembles allopregnanolone, and though it has not been studied in larger populations it has shown promising results. Indirect adjuvant treatments that have shown a more discrete efficacy include corticosteroids, which reduce the burden of anti-N-methyl-D-aspartate (NMDA) receptor antibodies in addition to their well-known anti-inflammatory properties. Stiripentol, normally used to treat Dravet syndrome, causes a diminution in seizure threshold through allosteric modulation of GABA. There are still gaps in our knowledge of the physiopathology and treatment of PCDH19, and large population studies are needed to make recommendations for proven therapeutic approaches for these patients.
